# Evaluation of the effectiveness and cost-effectiveness of psychological first aid (PFA) system based training for frontline health workers in emergency health services in China a study protocol of a cluster randomized controlled trial

**DOI:** 10.3389/fpsyt.2022.1044594

**Published:** 2022-12-16

**Authors:** Min Peng, Tao Xiao, Ben Carter, Pan Chen, James Shearer

**Affiliations:** ^1^King’s Health Economics, Institute of Psychiatry, Psychology and Neuroscience, King’s College London, London, United Kingdom; ^2^Psychological Rescue Branch of China Association for Disaster and Emergency Rescue Medicine, Changsha, China; ^3^Department of Social Affair, The Second Xiangya Hospital of Central South University, Changsha, China; ^4^Biostatistics and Health Informatics, Institute of Psychiatry, Psychology and Neuroscience, King’s College London, London, United Kingdom; ^5^Hunan Cancer Hospital, The Affiliated Cancer Hospital of Xiangya School of Medicine, Central South University, Changsha, China

**Keywords:** trial cost-effectiveness analysis, effectiveness evaluation, study protocol, psychological first aid training, frontline health workers, emergency health services

## Abstract

**Introduction:**

There is insufficient evidence on the effectiveness and cost-effectiveness of psychological interventions to enhance frontline responders’ mental health proficiency and competence in emergency settings. This paper describes the methods for the evaluation of the effectiveness and cost-effectiveness of Psychological First Aid training, to determine whether the intervention is effective and cost-effective as a mental health intervention in public health emergencies. A pilot to evaluate the acceptability of the PFA training found participants were either satisfied (55.6%) or extremely satisfied (44.4%) with the training program.

**Method:**

This paper describes the protocol for a cluster randomized two-arm controlled trial. A total of 1,399 non-specialist health care workers will be recruited in 42 hospitals and health care centers across six provinces in China. Participants will be assigned according to hospital or health care center site to one of two groups (*n* = 699 for intervention group and 709 for control group) to receive system based PFA training or PFA training as usual. Both groups will receive one-day of training, comprising six modules including PFA core concepts, knowledge, skills and practice. Their knowledge, skills, competency, self-efficacy, resilience, and professional quality of life will be assessed immediately after the training; and reassessed after 1 and 2 months.

**Analysis:**

For effectiveness outcomes, repeated measures will be used in a multi-level linear mixed model. The pooled standard deviations will be used to calculate the effect sizes (Cohen’s d) within and between groups. Appropriate statistical tests will be used to explore differences between intervention and control groups. For economic outcomes, a health service sector perspective will be adopted, with intervention costs and outcomes collected prospectively. Within-trial cost-effectiveness analysis (CEA) will quantify the incremental costs and PFA proficiency gains of the intervention compared with training as usual at 2 months post training. CEA will present results as cost per unit of mental health proficiency gained. A cost-utility analysis (CUA) model will extend the population to emergency health service users.in order to identify potential for incremental cost offsets attributable to mental health improvement arising from intervention. Intervention costs and effects will be extrapolated to the population of patients who receive the emergency health service in clinical wards and will be modeled over the cohort’s lifetime. Modeled CUA results will be calculated as quality-adjusted life-years saved and healthcare cost savings in preventing mental disorders.

**Ethics and dissemination:**

Ethics approval was obtained from the Second Xiangya Hospital, Central South University Clinical Research Ethics Committee (2021) Ethical Review [Clinical Research] #067). Data about the economic evaluation of the intervention will be stored in the KCL OneDrive at King’s College London, UK. The trial protocol was registered with the China Clinical Trials Registry: ChiCTR2200060464. Study findings will be disseminated through peer-reviewed academic papers, and participating hospitals.

## Highlights

This protocol has some limitations such as the absence of emergency health service users and social perspectives about time costs and outcome measures. However it contributes to the limited number of studies of evidence on the cost-effectiveness of interventions that improve mental health proficiency for frontline health care workers.

- Data collection for the economic evaluation is prospectively planned alongside the randomized controlled trial to assess intervention effectiveness.
- The cost-effectiveness study will generate important information for decision-makers, providing evidence of the “value-for-money” of the intervention.
- A modeled economic evaluation will estimate the long-term potential for the cost-effectiveness of the intervention as an emergency psychological preparedness and response method.

## Introduction

Failure to address mental health issues within populations recovering from crises including natural disasters or public health emergencies can further compound economic pressures and result in higher economic losses (1). An efficient response is pivotal to coping with distress and disturbance and ensuring the quality of mental health services. As the first emergency response, non-specialist health care workers’ capacity building is a critical issue for workforce resilience and a sustainable health system. These health care workers look after affected patients and are often exposed themselves to stressful and emotional situations; they may suffer ‘moral injury’ if forced to make decisions that don’t align with their values such as witness to intense human suffering and cruelty because of the lack of resources; or have increased anxiety about becoming affected themselves and/or passing infections or diseases on to their own families. Globally, Psychological First Aid (PFA) is recommended as an evidence-informed strategy for building mental health capacity (2). Some studies conducted in emergency humanitarian settings suggest that much of the heavy social burden is caused by associated mental health disorders which may be preventable through early intervention (3). The hypothesis of this study is that the mental health capacity of non-specialist health care workers can be strengthened through a system based PFA training program. As a result, health service users will benefit from the mental health service provided by the trained health care workers.

PFA can be used to train lay people as well as health care workers. It is an easy to implement strategy for non-specialist workers. The reason we chose health non-specialist workers is that we discovered there is acute lack of psychiatrists and mental health specialists to treat people especially in disaster and emergency settings. Health frontline workers treat physical injuries and illness as well as being the first contact to the survivors. They have the potential and clinical skills to be effectively trained as psychological first aiders as recommended by World Health Organisation (4). PFA has the potential to equip non-specialist health care workers with mental health service capacity, which can be integrated into existing specialist psychological support for emergency service users. Studies suggest a need to evaluate both PFA intervention’s effectiveness and the economic consequences. However, the research evidence on the effectiveness and cost-effectiveness of PFA training for non-specialist health care workers in emergency preparedness and response is scarce (5). Only a few high-quality studies have directly evaluated psychological intervention by non-specialist healthcare workers to alleviate impacts on acute and long-term psychological well-being (6, 7). The proposed study will firstly: test the effectiveness of the adapted PFA training program for enhancing non-specialist health care workers’ mental health capacity; and secondly, measure the cost-effectiveness of the training for trainees to improve their PFA profieciency.

The Chinese PFA training program is based on a PFA Train the Trainer (ToT) manual adapted by the Psychological Rescue Institute of China Association for Disaster and Emergency Rescue Medicine (8), which includes elements of mental health awareness along with PFA training based on the Johns Hopkins University PFA guidelines (9), It is a system based program which is an integrated mental health training model. The system based training model mainly includes PFA core conception and theory courses, and practice, adding WHO guidelines, and current policies. We will compare training as usual to the CPFA system based training program. The training as usual courses are mainly based on the Mental Health Gap Action Program (mhGAP) Intervention Guide (IG) in non-specialized health settings (10) since its efficacy is already well established in the literature (11). The PFA system based training model involved six modules including PFA core conception, knowledge, skills and practice. The system based training model has been incorporated into the National Emergency Assistance Work Guideline by National Health Commission of China for training frontline responders to provide emergency psychological assistance for people affected by natural disasters and public health emergencies. The work guideline book will be published in 2022 by People’s Medical Publishing House (Accepted at Jan 13, 2022, and in press).

## Aim and objectives

### Research question

Will the PFA system based training program be effective and cost-effective in terms of the primary outcome of change in non-specialist health workers’ mental PFA proficiency, and secondary outcomes of self-efficacy, confidence and professional quality of life at two months post training?

### Hypothesis

Non-specialist health workers in the system based training arm will show superior improvement in the primary outcome in non-specialist health workers’ mental PFA proficiency. The intervention arm will also show favorable cost effectiveness.

### Objectives

The project’s overall aim is to improve the capacity of non-specialist health care workers in China to provide mental health services by applying an integrated mental health training model and thereby ultimately improve the mental well-being, resilience and health related quality of life of emergency service users.

(1). To test the effectiveness of non-specialist health care workers trained by the CPFA training program in improving their mental health capacity;

(2). To test the cost-effectiveness of a system based training model to deliver training in the Chinese PFA to build mental health capacity in non-specialist health care workers.

## Study design, participants, and setting

### Design

The study will be a two-arm, participant-blinded, cluster randomized controlled trial, comparing PFA system based training with training as usual (1:1) to improve to improve the mental health capacity of non-specialist health care workers in China over two months (eight weeks).

### Setting and participants

Hospitals will be chosen to ensure sufficient number of clusters. In China, we contacted 73 public hospitals and health centers in six provinces of Hunan, Hainan, Xinjiang, Fujian, Dalian, and Jiangxi, presented the study to their staff members, and invited them to participate. Forty-two agreed to participate. The randomization was at the hospital cluster-level which to ensure minimal contamination. The hospitals will be randomly allocated to one of two groups (the PFA training group or the control training as usual group) with a ratio of 1:1 with stratification by hospital type (primary, secondary). The randomization will be conducted by an independent administrator at CADERM not associated with the study using a block-randomization schedules provided by the study statistician. The potentially eligible subjects will be invited to participate via a text message after informed consent is obtained from all participants.

### Eligibility

Inclusion criteria will be frontline doctors and nurses who voluntarily participate, agree to sign informed consent, and who are not specialized in mental health in clinical work (e.g., emergency department, infectious disease department, orthopedics department, emergency unit, etc.). All individuals who meet the inclusion criteria will be invited to participate, unless they are excluded due to previously receiving PFA training or training with overlapping content or physical illness.

### Ethical considerations

Our research will guarantee the confidentiality and safety of participants. We will not share personal information to any third parties, such as agents, employers without their consent. Informed consent will be obtained from all the participants. The researcher will provide information about the study and it’s aims to the participants before obtaining their informed consent. This study protocol has been approved by the Second Xiangya Hospital, Central South University Clinical Research Ethics Committee (2021) Ethical Review [Clinical Research] #067), and the trial protocol was registered with the China Clinical Trials Registry: ChiCTR2200060464^1^. Participants will provide written consent before the study begins. Participants can withdraw from the study at any time and for any reason without prejudice.

### Sample size

The total sample size of 1399 health care workers was estimated with a power = 0.80 and an alpha = 0.05, using PASS software based on *d* = 0.43 (12) and an ICC of 0.2 estimated in a similar study conducted in Nepal (13). The total minimum required sample size was 21 clusters per arm with 30 participants with an additional 10% inflation for drop out in each cluster to show a 20% difference in mean competency score on the psychological skills and knowledge (14) between the intervention group and control group immediately after training and at 1 and 2 month the follow-ups.

### Study procedure

**FIGURE 1:**
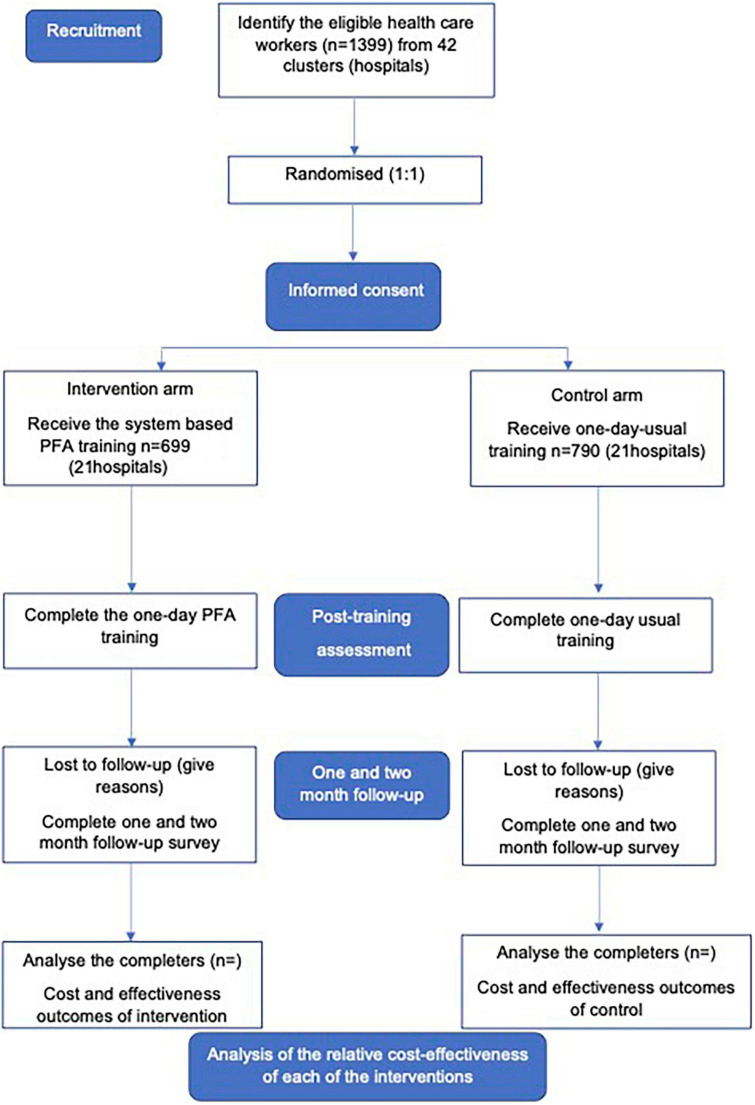
Design and flow chart of participants through the trial.

Participants will receive face-to-face or online one-day training intervention including six sessions, with follow-up assessment at 1 and 2 months after training. Before the training, participants will be selected for study eligibility according to the study’s inclusion and exclusion criteria. They will be evaluated by a research staff blinded to the intervention in each hospital. After the selection, participants will be assigned to one of the two groups and informed by message about their training (place and time). Groups of participants will receive training according to their random assignment group. There will be three assessment points for the intervention and control groups: (1) immediately post training, (2) one month post training, and (3) two months post training. Assessments will be undertaken remotely, with online instruments. An overview of the protocol procedure is provided in Figure 1.

### Randomization and allocation concealment

After the initial screening, 1,399 health care workers within 42 hospitals will be randomized in a ratio of 1:1 to either system based PFA training (intervention arm) or training as usual only (control arm). A random sequence created by the study statistician using varying block size (2 or 4) randomization technique. 21 hospitals will be allocated to the PFA system training group and 21 to the training as usual group by a senior administrator not involved in the study. Health care workers from each trial hospital will subsequently be assigned to either the control or intervention group along with their hospitals to avoid contamination. The number of participants for each trial cluster (hospital) will be approximately 33. The random allocation of the hospitals will be sent independently to the co-investigators’ email address. The email will not be sent to each study site until interventions are assigned.

### Blinding

Study participants are blinded while the trainers are not blinded, however the outcome assessors will not get involved in the training and are blinded to group assignment. The assessors and statisticians of the study will be blinded throughout the procedure and will not anticipate which arm the participants will be assigned. Outcome data provided to the statistician will be deidentified.

The key researcher (MP) will not have access to any identifiable data provided by health care workers participating in the RCT and the RCT dataset will be fully anonymized. Furthermore, the participating hospital’s identity, which could be used to identify health care workers, will also be fully anonymized.

### Interventions

The system based PFA Training model has six modules including core concepts, knowledge and practice. The training model has been incorporated into a National Emergency Assistance Work Guideline (in press) which has been adopted by National Health Commission of China to prepare the front-line non-specialist health workers in emergency settings. A pilot of the training intervention found it to be feasible for the paraprofessionals. The PFA training course was culturally tailored and localized based on a PFA ToT manual which included elements of mental health awareness alongside PFA training based on the Johns Hopkins University PFA guideline (9).

The PFA training program consists of one-day sessions comprising lectures, practice, and group discussions between lectures. All training sessions will be organized by a group of three trainers (from CADERM) who meet the following selection criteria: experience using psychological interventions, excellent teaching and communication skills, and willingness to conduct training of practitioners. They attended a 2-day face to face ‘train the trainer’ session delivered by a licensed psychiatrist from the Second Xiangya Hospital with over ten years of clinical psychological intervention practice. Additional training support for these trainers included 1. a teleconference facilitated by experts from Psychological Rescue Branch of CADERM to receive advice, support, and feedback on running participants’ training; 2. The licensed psychiatrists attend participants’ training sessions facilitated by the trainers to provide support and feedback monthly.

Participant training consists of PFA intervention knowledge, skills, and self-care. During the training, health care workers will learn skills and knowledge to (1) identify mental health need surging in emergencies (2) assess the psychological need, (3) refer to special care, and (4) self-evaluate and self-care. During the first four sessions, participants will be instructed in the basic concepts in psychological first aid and key principles, signs of stress and traumatic reactions, and key components of PFA. The participants will carry out group discussions around a case study in emergencies and disasters. During the last two sessions, participants will be instructed to practice a PFA intervention in an emergency. To ensure participants’ adherence, we assigned a facilitator to each training site to encourage them to practice and attend the discussions.

Because of its brevity and specificity to healthcare workers, the PFA training does not train the healthcare workers to identify mental disorders. Still, they will be able to refer to appropriate mental health services. Only about 20 min of the entire content is spent explaining differences between broad psychiatric groups, such as mood versus psychotic illnesses. Instead, participants are taught to assess for general signs that a person might be developing a mental health problem interfering with their usual activities. As such, no hypotheses about changes in participants’ rates of problem recognition are made.

## Training as usual

The participants in the control arm will receive usual training delivered from the same training staff. The materials are based on the Mental Health Gap Action Program (mhGAP) Intervention Guide (IG) in non-specialized health settings (10) and its efficacy was already well established in the literature (11).

## Acceptability and fidelity

### Pilot of PFA training

Between June and August 2022 we conducted a pilot study in the Second Xiangya Hospital, a tertiary hospital located in Changsha, the capital city of Hunan province. This hospital was not in the cluster study. The aim of this pilot study was to evaluate the acceptability of the PFA training. A convenience sample of non-specialist health care workers in the Second Xiangya Hospital (*N* = 18) participated in PFA training based on a professional continuing education one-day format. Acceptability was measured by health care workers-perceived ease of use, importance, and intention for follow up.

Most participants were satisfied with the training given by the trainers (55.6% strongly agree), the structure and content of training (66.7% agree and 33.3% strongly agree) and had no problems in participating the on-line training sessions (55.6% agree and 27.8% strongly agree). Furthermore, most of the participants agreed that their participation in the PFA training was voluntary (50% agree and 50% strongly agree).

Overall, participants were either satisfied (55.6%) or extremely satisfied (44.4%) with the training program, with all providing positive feedback about the pilot intervention: “It helped me cope the emergency situations of my daily work. I felt stronger and more confident to provide emergency mental health service for the patients.” None of the participants thought that the training program had negatively affected them. Cronbach’s Alpha for the 10-items was high (α = 0.881) indicating strong internal reliability of the questionnaire. Participants completed the questionnaire after they had successfully passed a practical assessment of their PFA skills given by the course trainer.

### Fidelity of intervention training

Quality and fidelity are required for ensure the successful delivery of training interventionto the participants as planned (15). Fidelity will be checked in both groups. The train the trainer sessions took place in person before the trainee participants were enrolled. Interventionists (trainers) were given standardized manuals that contained the same information about the PFA theoretical foundations. Three trainers and eight facilitators were trained by one supervisor, a senior clinical psychiatrist with over ten years of experience. The training focused on promoting consistency and reliability across all the delivery sites with specific attention to the scripts and standardized content. During the implementation of the training interventions, supervision occurs at weekly intervals for trainers. Initial training sessions will be recorded for use in monitoring fidelity in both groups. See Tables 1, 2.

**TABLE 1:** Fidelity checklist (Intervention arm).

Dimensions	Attributes of good practice	Questions for critical appraisal
I1: Statement of foundations	There should be a clear statement of the key concepts and principles of PFA The objective of the course should be defined The primary recipients of PFA should be stated clearly	Is there a clear statement of the key concepts and principles of PFA? Is the objective of the course specified and consistent with the stated concepts and principles of PFA?
I2: Statement of application/perspective	The primary recipients of PFA should be stated clearly The application of the training (conditions and timing) should be stated clearly and the application should be consistent with the stated principles and overall objective of the training The application of PFA should be specified and justified The outcome of the training should reflect the application/perspective of PFA and should be consistent with the objective of the training	Are the primary recipients specified? Is the application of the training consistent with the stated principles? Has the application of the training been stated and justified? Are the outcomes of the training consistent with the perspective, application and overall objective of PFA?
I3: Rational for the structure	The structure of the training should be consistent with a coherent theory of the PFA, and the crucial steps should be followed to implement the process of PFA intervention in disasters and emergencies All sources of evidence used to develop and inform the structure of the training (psychological first aid theory) should be described. The structure should be consistent with the objective of the training.	Has the evidence regarding the training structure been described? Is the structure of the training consistent with a coherent theory of PFA intervention in disasters and emergencies? Have any competing theories regarding PFA structure been considered? Are the sources of evidence used to develop the structure of the training specified?
I4: Strategies/techniques	There should be a clear definition of the strategies in PFA	Is there a clear definition of the strategies of PFA? Have all feasible and practical options been introduced? Is there justification for the exclusion of feasible options?
Safety and Comfort Stabilization Information Gathering: Current Needs and Concerns	The key strategies incorporated in the training should be described and the source of references should be given in detail to allow the trainees to be aware of the strategies that have been incorporated	Is the strategies incorporated into the model appropriate? Is the source for the strategies referenced? Are the methods for the strategies justified? Have the strategies incorporated into the training been described and referenced in sufficient detail?
Taking care of yourself	Individual self-care Reflect on own practice Protect yourself	Is the process of self-evaluation and self-caring clearly described? Have the method and resource been described in sufficient detail?
Practical Assistance Transfer and linkage with collaborative services	To offer practical help to survivors in addressing immediate needs and concerns	Is the logic of the PFA practice has been explained and justified?
	To link survivors with available services needed at the present time or in the future.	Have any differences between PFA and other interventions been explained?

**TABLE 2:** Fidelity checklist (Control arm).

Dimensions	Attributes of good practice	Questions for critical appraisal
C1: Statement of foundations	There should be a clear statement of the key concepts and principles of the general mental health training	Is there a clear statement of the mental health concepts and training content?
C2: Statement of application/perspective	The application of the common psychological treatment and mental health interventions (conditions and timing) should be stated clearly and the application should be consistent with the stated principles and overall objective of the training	Are the primary recipients specified? Is the application of the training consistent with the stated principles? Has the application of the training been stated and justified? Are the outcomes of the training consistent with the perspective, application and overall objective of training?
C3: Rational for the structure	The structure of the training should be consistent with a coherent theory of the mhGAP, and the guideline should be followed to implement the interventions The structure should be dictated by current patterns of service provision All sources of evidence used to develop and inform the structure of the training (i.e., the theory of behavior based treatment) should be described. The structure should be consistent with the objective of the training.	Has the evidence regarding the training structure been described? Is the structure of the training consistent with a coherent theory of psychological therapy and mental health interventions in disasters and emergencies? Are the sources of evidence used to develop the structure of the training specified?
C4: Strategies/techniques	The training should include possible options that represent current good practice Trauma rehabilitation interventions in disasters and conflicts Psychological debriefing or critical incident stress debriefing (CISD)	Are there clear definitions of incorporated techniques? Have all feasible and practical options been introduced? Is there justification for the exclusion of feasible options?
Balint groups for medical practitioners	To help medical practitioners to reflect their practice and reconsider the emotional relationship with the patients	Have the Balint group process and techniques been described and referenced in sufficient detail?

Outcomes.

### Primary outcome

#### PFA knowledge, skills, and attitude

The primary outcome of the training is to facilitate non-specialist health care workers with the necessary knowledge, skills, and motivations to recognize a mental disorder presented in a vignette using the SKA-CPFA developed by licensed psychologists from CADERM (16). Post-test questions for PFA knowledge and skills in Chinese are also provided. Professional attitude will be assessed with a 20-item questionnaire with a score from one to five. Items are scored on a 5-point scale (1 = strongly agree; 5 = strongly disagree), and the total score ranges from 20 to 100. The higher the score, the more positive the gained capacity. The items are characterized in three domains: 1. how well the participants communicate their understanding of knowledge of PFA content (40 points); 2. skills related to the psychological intervention (including self-efficacy, proficiency, and self-assessment of relevant skills for the implementation of PFA) (30 points); 3. Attitudes including perception of barriers and personal judgments about the value of emergency psychological intervention (30 points). The questions reflect the health care worker’s non-judgmental attitude toward providing mental health support to other people. Responses are close-ended, and the resulting data will be recorded by a researcher blind to the cluster allocation and in accordance with the protocol.

### Secondary outcomes

#### Professional quality of life

The Professional Quality of Life Scale (ProQOL-5) is a 30-item scale to assess one’s perceived quality of life about working as a helper and includes both the positive and negative aspects of this work. It is a 5-point Likert self−reporting scale and consists of three subscales of professional QoL: compassion fatigue (CF), burnout (BO), and compassion satisfaction (CS). For each of the sub-scales, scores are categorized as Low (22 or less), Moderate (between 23 and 41), or High (42 or more). With a low professional QoL, health workers may manifest a loss of self−worth and diminished productivity, and staff turnover can be affected.

#### General self-efficacy

The General Self-Efficacy Scale is a 10-item psychometric scale designed to assess optimistic self-beliefs to cope with difficulties in life. The scale was developed to assess optimism referring to the personal belief that one’s actions are responsible for successful outcomes. The total score is 10 to 40. Higher scores indicate higher perceived general self-efficacy, and lower scores indicate lower perceived general self-efficacy.

#### Positive psychological change after the experience of traumatic events

PTGI (Post-traumatic Growth Scale) is used to demonstrate positive outcomes of the emergency events; for example, health care workers show improved psychological functioning in specific domains and a sense of increased job competency and confidence. The PTGI consists of 21 items, each rated on a 6-point-Likert scale (ranging from 0 = I did not experience this change due to my crisis to 5 = I experienced this change to a very great degree as a result of my crisis). In each of the five domains, changes may occur at the affective, cognitive, and behavioral levels. The mean PTG score reported of 55.94 was considered a moderate level of PTG. A similar mean score of 51.36 (SD = 19.90) was reported in Jordan and in Turkey (*M* = 49.11, SD = 29.11).

### Economic measures

#### Resource use

Key resource use and costs will be estimated based on the health provider perspective, which means the relevant costs including time spent by individuals on developing or delivering the trainings such as mental health professionals and training management (administrators, team members, psychiatrists, psychologists, and training assistants) and supervision. In addition, the time and costs associated with the training development, consumables/materials costs, travel cost, trainers’ wages and trainees’ time costs Cost and effect data are collected within a 12-month time horizon and were therefore not discounted.

#### Costs

Intervention costs will be estimated using bottom-up micro-costing and include labor costs of the program trainers, staff training costs, material costs, travel costs, and accommodation costs (17). Labor costs are based on the local average wage of hour per program meeting per program trainer (6 h in total for PFA and 4 h for control). Labor, training, managerial, and travel costs were based on an average time investment of per training session per session trainer. Training costs were based on full staff compliance with the program (16 h for clinical health care workers, 16 h for training management workers). Labor and training costs are valued using gross hourly wages. Costs will be expressed in 2021/2022 RMB yuan (¥). A detailed list of relevant resource use categories, sources of usage data, and unit costs are given in Table 3.

**TABLE 3:** Resource use and intervention costs.

Cost item	Source	Unit cost	Calculation
**Pre-training costs**			
Curriculum design	Trial budget	¥ 500 per session	No of sessions *X* value of the course
Materials: participant manuals	Trial budget	¥ 50 per person	No of manuals = total number of participants (1260 + 79 + 60 = 1399)
**Train the trainer training venue**			
Instructor	Trial Expert Time Use Records	¥ 1000 per day	No of training days × value per day
Meals and tea break	Trial budget	¥ 50 per person	No of trainers × value per person
Trained trainer (Clinical psychologists)	Trial budget	¥ 60 per hour	Hours of work leave × daily wage
**Train the trainees training venue**			
Online Training Equipment	Trial budget	¥ 500 per day	No of days × price per time
Trained trainer (Clinical psychologists)	Trial budget	¥ 60 per hour	Hours of work leave × daily wage
Training assistant	Trial budget	¥ 200 per person-day	days of work × unit wage
Hardcopy of certificates for the trainees	Trial budget	¥ 50 per person	No of participants x (certificate cost + delivery fees)
**Indirect costs**			
Absenteeism	Ministry of Human Resources and Social Security of China “Enterprise salary survey information in 2020”	Attendee number and their daily wages according to profession classification assigned (Chinese National Ministry of Finance)	Days of work leave × daily wage

Healthcare costs will be estimated using tariffs from the Chinese Health Statistical Bureau.

Data on willingness to pay for the training program are collected by asking participants the question “Would you pay (about as RMB of 2022) to participate in the training program?”. The amount RMB was determined based on local cost information. Once the training materials are prepared, and the training contents are standardized, it may be possible to reduce the cost of the training program per participant to this level in the future. This hypothetical question is not accompanied by any actual payment for a training fee and does not penalize respondents who give an affirmative answer. The willingness-to-pay question is hypothetical also in that when the question is asked of the training participants during the follow-up surveys it will be whether they would pay to participate in the training program if they had not received the training.

However, this question may overestimate the importance of involvement in training. Blumenschein (18) performed laboratory tests to find a way to eliminate such bias in replies to hypothetical questions in general and concluded that the “certainty approach” can reduce the prejudice to a negligible degree. We use this strategy, which is the same as asking an additional question, “How confident are you about the response?” if the above willingness-to-pay question was positive, with dichotomous options of “absolutely sure” or “probably sure,” and only counting “certainly sure” as the affirmative answer.

### Analysis

#### Data analysis

Statistical analysis will be performed using STATA (version 17 or later) and a *p*-value < 0.05 (two-tailed) will be taken to indicate statistical significance.

All trial participants who were randomly assigned at the start of the trial will stay in the sample for analysis under the intention-to-treat principle. It will be decided whether the intervention program is effective using random effect mixed modeling described below. Demographic factors will be evaluated for their potential influence on primary and secondary outcomes, and in cases where they do, they will be used as covariates.

The primary outcome for the statistical analysis will be the mean change in PFA-SKA score after 2 months of follow-up compared between intervention and control arms. The analysis allowed for this clustering in order to obtain unbiased estimation of the treatment effect and its standard error (SE). A two-level hierarchical mixed-effects model will be used to be fitted in the statistical analysis plan.

All secondary outcomes will be summarized by trial allocation group and follow-up period using mean, SDs and 95% CIs for the variables (ProQOL, GSE, PTGI scores). Statistical tests of differences for secondary outcomes will be performed using the same hierarchical mixed-effects model as the primary outcome analysis. In addition, participant-scores are also compared as change from post-training to 2-month follow-up using independent group t-tests.

#### Repeated measures modeling

For the primary and secondary outcomes, repeated measures will be used in a linear mixed model. ANOVA with time (T0-T1-T2) as a within-groups component and treatment condition as a between-groups factor will be utilized. Mixed-model repeated measures ANOVA does not substitute missing values; it uses all the data for each participant that are available. We’ll run a sensitivity analysis to examine the effect of drop-outs on our findings. The full data sets of participants who finish all sessions of the training will be the basis for the protocol analysis. The time to onset of clinical outcomes will be based on post-training interview and two follow-up assessments. The pooled standard deviations will be used to calculate the effect sizes (Cohen’s d) within and between groups. Appropriate statistical tests will be used to explore differences between intervention and control groups (Fisher’s exact test as appropriate). The nature of the routine administrative data will determine the test’s selection. SPSS statistics, Excel, and Visual Basic for Applications will be used for all analyses (VBA).

The multilevel analysis will be used with nested data to account for clustering and adjust for the type of critical incident, age, and sex. Means and standard deviations for continuous outcomes and proportions for categorical outcomes will be reported.

#### Populations under investigation

Any participant with any post-baseline follow up will be included in the modified intention to treat (ITT) population. We will remove the missing data from analyses using listwise or pairwise deletion. A multilevel multivariable linear model does not substitute missing values; it uses all of the data for each participant that are available. The full data sets of participants who finish all sessions of the training and respond to the first round interview will be the basis for the protocol analysis.

#### Within-trial cost-effectiveness analysis

The within-trial economic evaluation will assess cost per unit improvement in skills knowledge for the non-specialist health care workers with methods reflecting good practice guidance and methodological recommendations by ISPOR. The primary economic analysis will take a Chinese health system perspective. Uncertainty will be represented using incremental cost-effectiveness acceptability curves and net benefit analysis (19). The costs of the training intervention will be calculated based on hours that the designing, printing of the training materials, the hours of health care workers attending the training, the hours of the trainers delivering the training, plus travel fees and hours of preparation and supervision. Incremental cost-effectiveness ratios (ICERs) will be calculated as the difference in costs between the intervention and the comparator divided by the difference in benefit.

#### Modeled cost-utility analysis

The modeled CUA will assume that the intervention is integrated with the national curriculum and will include costs accumulated during the intervention implementation phase and effects over a longer time horizon, assumed to be rest-of-life or 75 years of age.

Monte Carlo simulation will determine the 95 percent confidence intervals around epidemiological probability and cost estimates using the Microsoft Excel add-in Ersatz38. Sensitivity analysis will be undertaken for the modeled CUA by varying key assumptions around the intervention effect on emergency health service users (patients). The model will use data from the Chinese Health Survey and disease epidemiology from the Global Burden of Disease study see Table 4.

**TABLE 4:** Input parameters for health impact modeling.

Parameters	Data source and assumptions
Total population estimates (population numbers, gender distribution)	CHARLS (China Health and Retirement Longitudinal Study)
Mental disease epidemiology, disability weights	National Health Information Statistic Bureau of China (http://www.nhc.gov.cn/mohwsbwstjxxzx)
Relative risks of mental health-related diseases by risk categories	
Relative risks, total years of life lived with mental disorders	
Disease healthcare costs	National Health Information Statistic Bureau of China (http://www.nhc.gov.cn)

### Data storage

All questionnaires in this study will be saved in the form of network data. All electronic files and data are only stored in the research team’s locked computer to protect the privacy of the respondents. The data will be fully anonymized after analysis and participants will not be identified in any publications.

### Timeline

#### Date of trial end

The trial will end when the last participant accomplished the two-month post-training interview.

### Training of assessors

Before the questionnaire survey, the research team assessors of this study will be trained to ensure that the assessors have fully understood the main contents of the questionnaire set.

### Measures to improve compliance of respondents

Researcher shall implement informed consent, ensuring the respondents fully understand the research requirement and be willing to be followed up.

## Strengths

Data from the CEA within the study will be augmented with results from a modeled CUA, which will look at the intervention’s overall cost-effectiveness by extending costs and effects to patients and modeling over a longer time horizon. The proposed economic evaluation will add to the relatively limited data base on the “value for money” of mental health interventions in emergencies. It will provide useful information to healthcare decision-makers on the economic justification for more widespread deployment.

## Limitations

A potential limitation of the study may be missing data on social perspectives about time costs and outcome measures.

## Author contributions

MP and JS: conception of the study. BC: method and design. TX: clinical registration. MP and PC: drafting the manuscript. All authors contributed sufficiently to the manuscript to be included as authors.

## Footnotes

1
https://www.chictr.org.cn
